# A multilingual, multi-country dataset on gardening and biodiversity awareness across Central and Eastern Europe

**DOI:** 10.1038/s41597-026-07887-9

**Published:** 2026-07-31

**Authors:** Zsófia Varga-Szilay, Lucija Šerić Jelaska, Silva Vilumets, Arvīds Barševskis, Klára Benedek, Danilo Bevk, Agata Jojczyk, Anton Krištín, Jana Růžičková, Eve Veromann, Kinga Gabriela Fetykó, Gergely Szövényi, Gábor Pozsgai

**Affiliations:** 1 HUN-REN-PE Evolutionary Ecology Research Group, University of Pannonia, Veszprém, Hungary; 2 Dr. Franjo Tuđman Defense and Security University, Zagreb, Croatia; 3 Chair of Plant Health, Institute of Agricultural and Environmental Sciences, Estonian University of Life Sciences, Tartu, Estonia; 4 Coleopterological Research Center, Institute of Life Sciences and Technology, Daugavpils University, Daugavpils, Latvia; 5 Department of Horticulture, Faculty of Technical and Human Sciences, Sapientia Hungarian University of Transylvania, Târgu-Mureș, Romania; 6 National Institute of Biology, Biotechnical Faculty, University of Ljubljana, Ljubljana, Slovenia; 7 Department of Landscape Architecture, Institute of Environmental Engineering, Warsaw University of Life Sciences, Warsaw, Poland; 8 Institute of Forest Ecology, SAS, Zvolen, Slovakia; 9 HUN-REN-ELTE-MTM Integrative Ecology Research Group, Budapest, Hungary; 10 Department of Systematic Zoology and Ecology, ELTE Eötvös Loránd University, Budapest, Hungary; 11 Independent researcher, Kecskemét, Hungary; 12 Center of Applied Economic Studies of the Atlantic, University of the Azores, Angra do Heroísmo, Terceira, Azores Portugal

## Abstract

We present a multi-country and multilingual dataset on garden characteristics, gardening practices, and gardeners’ environmental attitudes based on a questionnaire survey conducted across nine Central and Eastern European countries: Croatia, Czechia, Estonia, Hungary, Latvia, Poland, Romania, Slovakia, and Slovenia. The survey was collaboratively designed and translated into ten languages by native speakers to ensure cultural and linguistic accuracy. It was distributed online through diverse channels, yielding 5511 responses. The dataset enables exploration of relationships between gardening practices and socio-cultural factors at local, national, and regional scales. The questionnaire covered topics such as garden characteristics, management practices, pesticide use, biodiversity-supporting actions, and environmental awareness, offering a comprehensive overview of gardening in the region. The dataset is suitable for cross-country comparisons, machine learning analyses, and the development of indices that quantify ecological value or management intensity. The multilingual questionnaire supports reuse in future studies, facilitating replication or adaptation elsewhere. By making the dataset openly available, we provide a resource for researchers and practitioners interested in urban biodiversity, sustainability, and human–nature interactions.

## Background & Summary

Urbanisation is a major driver of biodiversity loss worldwide^1,2^, contributing to habitat fragmentation^3^, the disruption of ecological interactions^4^, and the decline of ecosystem services essential for human well-being, such as pollination and natural pest control^5^. To mitigate these impacts, attention has increasingly turned to enhancing the ecological quality of urban green spaces, especially in densely human-modified environments like cities^6,7^. While public parks and other green infrastructure are often prioritised in conservation planning^8,9^, private domestic gardens and allotments also represent a substantial proportion of urban land cover in Europe — estimated between 16 and 36% depending on the country^10,11^ — and therefore their role in urban biodiversity and ecosystem services should not be overlooked. These privately or small community managed spaces can provide habitats and stepping stones for wildlife, improve ecological connectivity, and deliver multiple benefits for biodiversity and people alike^12,13^.

The conservation potential of private gardens and allotments depends strongly on how they are managed^14–16^. Practices such as frequent mowing, pesticide use, or the removal of microhabitats reduce biodiversity, while creating undisturbed patches, planting native species, or maintaining ponds can significantly enhance it^15,17^. Consequently, understanding garden management practices is key to evaluating gardens’ contribution to the biodiversity of urban ecosystems^16^.

Research into the biodiversity of private gardens and allotments has expanded considerably in Western Europe and North America since the 1990s, with studies showing that even small gardens can support high levels of species richness^18^ and act as habitat refuges and stepping stones in urban ecological networks^19^. In contrast, comparable research in Central and Eastern Europe (CEE) remains limited, despite the region’s distinctive socio-economic context and gardening traditions^16,20^.

In Central and Eastern Europe, gardening—traditionally a means of food self-provisioning during economic hardship—has only recently shifted toward recreational and biodiversity-oriented forms, yet limited access to ecological knowledge and continued reliance on conventional, pesticide-based practices indicate slower adoption of biodiversity-friendly approaches^16^. These shifts highlight the need for CEE-specific data to better understand how cultural and historical background, gardening motivations, and socio-economic drivers shape garden management and ecological outcomes in the region and in countries showing similar trajectories.

This dataset^21^, available through the Figshare repository, aims to fill this information gap and gathers structured data on the gardening practices and gardening motivation of CEE countries. It provides anonymised data from a cross-national questionnaire survey of nine CEE countries. The survey, containing 59 questions, designed to collect detailed information about respondents’ socio-demographic background, personal motivations for gardening, and the characteristics of their gardens (e.g. its size, how much area was occupied by different types of plants, such as fruit trees, ornamental plants, and lawn, and what was the adjacent land use), as well as their plant protection and -growing practices. In addition, the survey recorded information on pesticide use habits (types of products used, target organisms, perceived importance of pesticide use, and purchasing habits, etc.), mowing frequency, biodiversity-supporting practices (especially for insect pollinators) and their practical implementation (e.g. creating supporting habitats), as well as on the degree of environmental awareness of the gardeners (e.g. opinions about threats to pollinators and knowledge of risks associated with synthetic pesticides, like neonicotinoids). Respondents were also asked about their familiarity with the biodiversity present in their gardens and the tools they use for species identification (e.g. whether they use mobile applications), as well as whether they apply spot-on insecticide treatments on their pets that are allowed outdoors in the garden.

A total of 5511 gardeners responded to the questionnaire, representing a wide range of cultural backgrounds and gardening practices. The data provide a valuable resource for investigating the interplay between socio-demographic factors, garden management practices, and gardener attitudes in maintaining biodiversity-friendly gardens across multiple scales and allows for investigations using a variety of analytical approaches, including complex statistical modelling, machine learning, and network analysis. Indeed, subsets of this dataset have already been used to investigate CEE gardeners’ attitudes towards biodiversity-friendly gardening and small-scale variation in these attitudes^15,16^. However, while the datasets underlying those studies partially overlap with the dataset described here, the present data descriptor provides the complete questionnaire and associated responses, including survey questions not analysed previously, as well as responses from participants who were excluded from the earlier studies. In addition, the entire questionnaire, including all questions and structure, is available in ten languages, enabling its reuse either in full or on a question-by-question basis.

## Methods

### Study area and data collection

The questionnaire was distributed in nine CEE countries (Croatia, Czechia, Estonia, Hungary, Latvia, Poland, Romania, Slovakia, and Slovenia), all of which were formerly part of the Eastern Block and are currently members of the European Union.

The questions were developed in Hungarian and translated first to English and then from English to Croatian, Czech, Estonian, Latvian, Polish, Romanian, Slovak, and Slovenian languages by native-speaking collaborators to ensure linguistic and cultural accuracy. Thus, the participants could choose from the ten languages in which they wanted to complete the questionnaire. The wording of all questions was kept deliberately neutral and simple to avoid bias and respondents were also able to contact local collaborators for clarification in their own language.

The questionnaire was distributed exclusively online and was made accessible in two ways: through a direct link to the Google Form(s), and via a dedicated website (https://vargaszilay.hu/questionnaire/) where respondents could select their preferred language from the available translations before completing the survey. The questionnaire was actively distributed for a 90-day period in each of the nine participating countries between 26 October 2022 and 18 May 2023, with the start and end dates slightly varying by country. Following this period, the survey remained accessible online but was no longer actively promoted. The released dataset contains responses from 26 October 2022 to 13 June 2023 time window. The questionnaire was disseminated through a broad variety of channels to reduce the risk of bias towards particular respondent groups. These channels included gardening-oriented websites, and various online social media platforms (for instance Facebook groups and pages) where QR codes and hashtags were used to increase the sharing efficiency. Moreover, through targeted email distributions, outreach efforts extended to professional associations, non-governmental organisations, foundations, and societies dedicated to gardening and environmental conservation. To standardise dissemination, a document with ‘Tips and Tricks’ was provided to collaborators, together with an English template text for social media sharing, which could be translated into their respective languages.

### Ethics statement

Ethical approval for this study, including the study protocol and data sharing, was granted by the Daugavpils University Biology and Medical Research Ethics Committee (No. 107). According to the European GDPR regulation, a Data Protection and Privacy Statement was available alongside the questionnaire in all languages. Thus ethical considerations were minimal and aligned with institutional protocols and the principles of the Declaration of Helsinki. Although, to a certain degree, our study involved humans, it did not constitute an experimental setting and did not involve the use of human tissue. Our online questionnaire was entirely voluntary and anonymous, and by completing the questionnaire, respondents consented to the use of their answers for research purposes. No personal data were collected, and before beginning, participants were informed about the aim of the study. The Form did not automatically store respondents’ email addresses; however, participants had the option to provide it at the end of the questionnaire or input personal information in the free text fields. This, and all other, potentially sensitive information was removed and pseudonymised (e.g. grouping participants within broad age groups rather than giving specific ages) prior to data release, ensuring that no respondent can be identified from the dataset in any form. Based on these, the Daugavpils University Biology and Medical Research Ethics Committee has thus approved a waiver of consent for the publication of the dataset (No. 107).

### Questionnaire structure

**Fig. 1:**
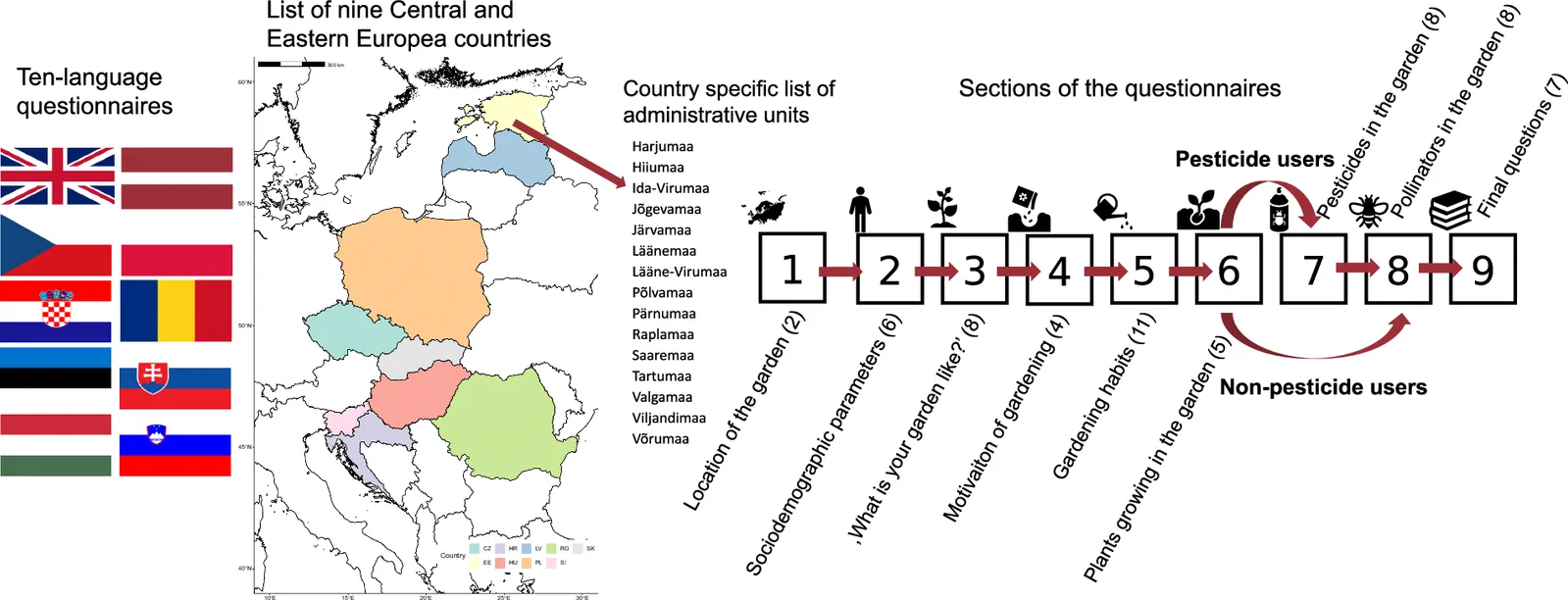
Structure of the multilingual, multi-country questionnaire. The numbers in parentheses after each of the nine section titles indicate the number of questions included in that section. Section 7 was available only to respondents who reported using pesticides at the last question of Section 6.

The questionnaire gathered information about the coarse location and the main characteristics of the garden, the sociodemographic parameters, the garden owners’ motivations, cultivation habits, pesticide usage, environmental awareness, and pollinator-friendly practices. The questions were organised into nine sections (1. Location of the garden; 2. Sociodemographic parameters; 3. ‘*What is your garden like?*’; 4. Motivation; 5. Gardening habits; 6. Plants growing in the garden; 7. Pesticides in the garden; 8. Pollinators in the garden; 9. Final questions, e.g. ‘*How did you learn about this questionnaire?’* or *‘How do you learn/gather knowledge/information about gardening?’*) (Fig. 1).

The questionnaire was designed in Google Forms, one form for each language, divided into separate pages to make completion easier for respondents, and took a maximum of 15 minutes to complete. It was mandatory to respond to 55 of the total 59 questions (see in the questionnaire.xlsx). Overall, we aimed to formulate the questions in a concise and straightforward manner. To facilitate easier data processing, free-text responses were not allowed, with the exception of two questions at the end of the questionnaire (‘*Please describe in a few words/one sentence what your garden means to you*.’; ‘*Please describe briefly what you like the most in your garden*.’). Thus, depending on the type of question, six different formats were used: checkbox (10), linear scale (5), multiple choice (26), multiple choice grid (14), paragraph (2), and short answer (1) (see details in the questionnaire.xlsx).

In the first two questions, the respondents could choose in which country and lower-level administrative units (respectively) their garden was located. The second section was country-specific: only administrative units of the previously chosen country were listed. We recorded the finer scale of residency according to the Nomenclature of Territorial Units for Statistics (henceforth NUTS^22^). Because of the large number of NUTS-3 regions in Poland, to simplify the questionnaire, here, residencies were recorded at NUTS-2 levels, while the other eight participating countries were recorded at NUTS-3 levels. After these, the questions were the same for everyone. However, section seven (Pesticides in the garden), which focused on pesticide-use habits, was shown only to respondents who answered ‘Yes’ to the question ‘*Do you use pesticides?*’. Respondents answering ‘No’ were automatically directed to Section 8 (Pollinators in the garden) (Fig. 1).

For the question ‘*What pollinators do you regularly observe/see in your garden?*’, photos were provided to illustrate insect pollinator groups (butterflies, bumblebees, honeybees, other wild bees, wasps, ants, hoverflies, other flies, and beetles). For the question listing potential threat factors and asking ‘*To what level do you think the factors below threaten wild pollinators?’*, respondents were explicitly informed that the domestic honeybee is not considered a wild pollinator.

### Data processing

The data were downloaded from the Google Forms as plain coma-separated text (.csv) files in each language by the first author, then, to improve the usability of the dataset and to allow future reuse, formatting and harmonisation processes were carried out in Python and R environments.

The data cleaning was performed in a Python environment^23^ using the ‘pandas’^24^ package. The figures in this paper were produced in an R environment^25^, using the ‘sf’^26^, ‘ggspatial’^27^, ‘rnaturalearth’^28^, and ‘ggplot2’^27^ packages. In addition, two shapefiles^29,30^ were used to support the spatial visualisations.

After selecting their country, respondents could choose the administrative unit where their garden is located (Fig. 1), as displayed in their respective language, including special characters, where applicable. Since these administrative unit names often contained special characters, we matched them to the corresponding NUTS categories for standardisation (see in the dictionary.xlsx).

To facilitate cross-linguistic comparability, and to avoid encoding incompatibility issues with special characters, both the survey questions and response options were translated into English using a dictionary that aligns the multilingual content with standardised English terminology. The English language datasets were then merged and converted into comma-separated values (.csv; see in the main_dataset.csv). The data dictionary and the list of questions and answers are available in Excel (.xlsx) format. All files follow a consistent naming convention and UTF-8 encoding to ensure accessibility and interoperability across systems.

## Data Records

### Data structure, description of data and documentation files

The released dataset (main_dataset.csv) is available in the Figshare repository^21^. It consists of 5512 rows (one header row and 5511 respondent records) and 101 columns (questions), values separated by commas. It includes only the core questions; optional items such as the respondent’s email address and open-ended, free-text questions were excluded. Except for the first two columns, which are named as ‘ID’ and ’Country_code’, the columns in the dataset (main_dataset.csv) are labelled with question identifiers ranging from ‘Q_1’ to ‘Q_99’ (since the answers to multiple choice grid were recorded as separate columns, the number of columns exceeds those of the questions). For questions that allowed multiple answers, the responses are listed in one value field, separated by semicolons.

In addition, a separate dictionary file (dictionary.xlsx) is provided for the translations of the questions and answers to all languages, along with a list of the questions and possible answer options in English (questionnaire.xlsx). These files are cross-referenced with each other and with the main dataset through common identifiers (e.g. ‘Main_dataset_ID’ column). The questionnaire.xlsx file indicates the structure of the questionnaire, showing which of the nine sections each question belongs to, the question type (e.g. multiple choice), and whether answering the question was mandatory.

## Data Overview

### Characteristics of the sample population

Across all countries, the proportion of female respondents was generally higher than that of males (Table 1). Most participants were between 26 and 55 years of age and held middle-level (secondary or high school) or postsecondary education (college or university level). The majority of respondents lived in towns or rural areas, while smaller proportions resided in capital cities or large urban settlements. Approximately half of the respondents reported having children.

**Table 1:** Sociodemographic characteristics of the study population (n = 5511).

		Country ISO code																	
		CZ		EE		HR		HU		LV		PL		RO		SI		SK	
Variables	Levels	n	%	n	%	n	%	n	%	n	%	n	%	n	%	n	%	n	%
Gender	Female	531	79.85	664	93.26	389	80.21	915	72.68	350	88.38	538	71.45	300	67.42	292	83.19	303	68.09
	Male	129	19.4	46	6.46	96	19.79	343	27.24	46	11.62	212	28.15	144	32.36	59	16.81	140	31.46
	Other	5	0.75	2	0.28	0	0	1	0.08	0	0	3	0.4	1	0.22	0	0	2	0.45
Age	18–25	34	5.11	4	0.56	17	3.51	50	3.97	7	1.77	105	13.94	29	6.52	10	2.85	10	2.25
	26–35	179	26.92	89	12.5	70	14.43	196	15.57	43	10.86	91	12.08	75	16.85	49	13.96	60	13.48
	36–45	216	32.48	181	25.42	132	27.22	334	26.53	63	15.91	213	28.29	163	36.63	95	27.07	137	30.79
	46–55	131	19.7	217	30.48	154	31.75	334	26.53	130	32.83	187	24.83	95	21.35	90	25.64	113	25.39
	56–65	59	8.87	166	23.31	85	17.53	180	14.3	120	30.3	82	10.89	51	11.46	67	19.09	80	17.98
	Over 65	46	6.92	55	7.72	27	5.57	165	13.11	33	8.33	75	9.96	32	7.19	40	11.4	45	10.11
Education level	Elementary	8	1.2	11	1.54	0	0	7	0.56	4	1.01	8	1.06	2	0.45	4	1.14	4	0.9
	Middle	237	35.64	178	25	172	35.46	346	27.48	78	19.7	177	23.51	123	27.64	92	26.21	135	30.34
	Postsecondary	315	47.37	459	64.47	258	53.2	822	65.29	278	70.2	514	68.26	244	54.83	232	66.1	267	60
	Postgraduate	105	15.79	64	8.99	55	11.34	84	6.67	36	9.09	54	7.17	76	17.08	23	6.55	39	8.76
Type of the urban settlement Having children	Capital	60	9.02	42	5.9	83	17.11	154	12.23	22	5.56	128	17	6	1.35	26	7.41	86	19.33
	City	68	10.23	28	3.93	71	14.64	238	18.9	41	10.35	106	14.08	80	17.98	21	5.98	22	4.94
	Town	126	18.95	122	17.13	130	26.8	342	27.16	56	14.14	279	37.05	61	13.71	52	14.81	62	13.93
	Countryside	400	60.15	279	39.19	193	39.79	511	40.59	206	52.02	231	30.68	293	65.84	206	58.69	265	59.55
	Farmland	11	1.65	241	33.85	8	1.65	14	1.11	71	17.93	9	1.2	5	1.12	46	13.11	10	2.25
	Yes	385	57.89	378	53.09	248	51.13	615	48.85	229	57.83	396	52.59	251	56.4	187	53.28	267	60
	No	280	42.11	334	46.91	237	48.87	644	51.15	167	42.17	357	47.41	194	43.6	164	46.72	178	40
**Total number of respondents**		**665**		**712**		**485**		**1259**		**396**		**753**		**445**		**351**		**445**	

**Fig. 2:**
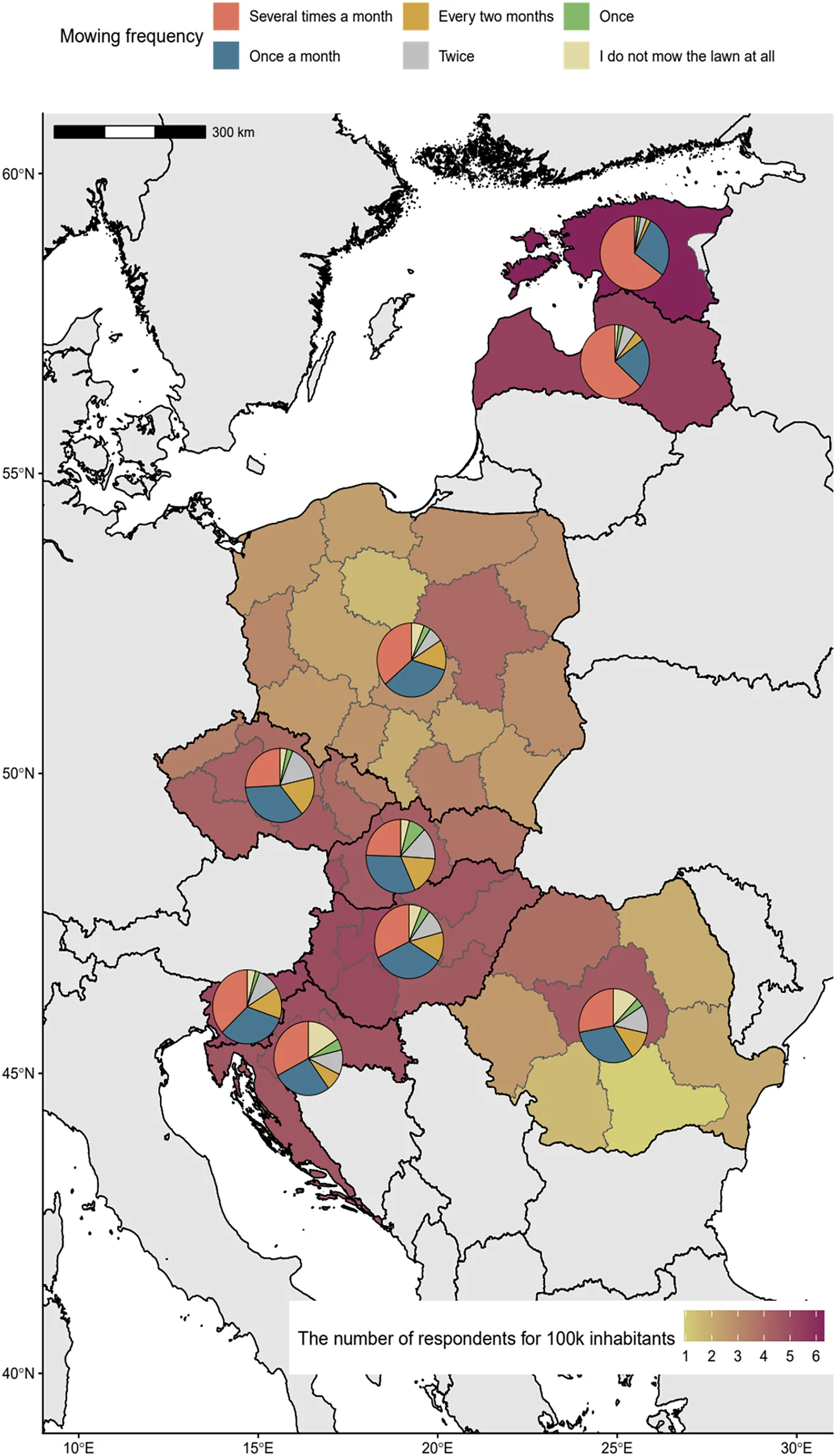
Number of respondents in the nine participating countries by NUTS-2 regions (thick black lines indicate national borders). The colour intensity of the maps represents the number of respondents per 100000 inhabitants in each NUTS-2 region and is displayed on a logarithmic scale. The pie charts within countries illustrate mowing frequency during the growing season (April–September). Different colours indicate the mowing frequency categories as follows: salmon – ‘Several times a month’, blue – ‘Once a month’, gold – ‘Every two months’, grey – ‘Twice during the growing season’, green – ‘Once during the growing season’, and pale yellow – ‘I do not mow the lawn at all’.

The largest number of respondents were from Hungary, and Estonia was the most represented country in proportion to its population size. Gardeners from certain regions in Romania and Poland showed relatively low willingness to participate in the survey (Fig. 2).

In terms of mowing practices, respondents from the two northernmost countries (Estonia and Latvia) reported mowing their lawns very frequently (several times per month) whereas in Croatia and Romania a notable share of respondents stated that they do not mow their lawns at all. Overall, mowing once a month was the most common frequency during the growing season, from April to September (Fig. 2).

**Fig. 3:**
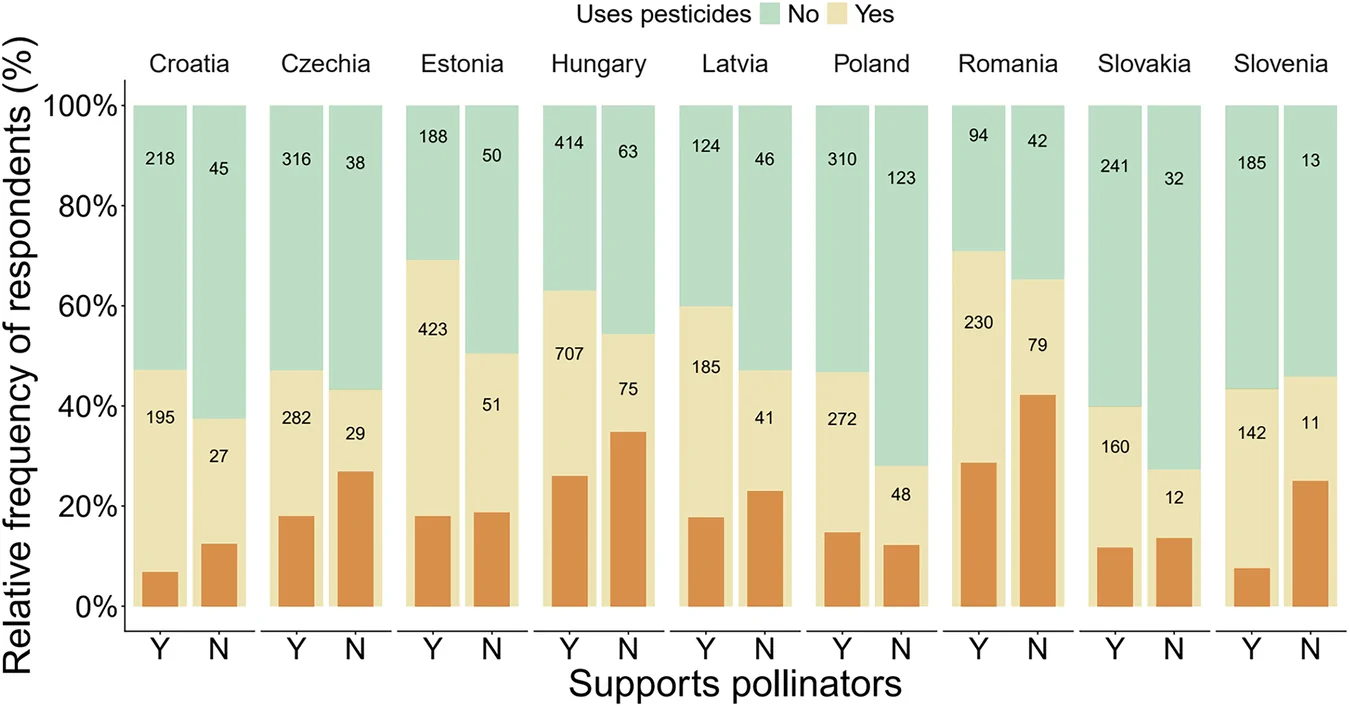
Relative frequency of respondents (gardeners) by whether they support pollinators (Y – Yes, N – No) and whether they use pesticides (No: light green, Yes: light yellow). Numbers within bars indicate the absolute number of respondents. The dark orange segments indicate the proportions of synthetic pesticides users within the pesticide-user groups.

In most countries, respondents who reported supporting insect pollinators in their garden were more prevalent than those who did not (Fig. 3). However, a relatively high proportion of pollinator supporters also reported using pesticides, and their pesticide use rate was higher compared to respondents who did not support pollinators. In contrast, respondents who did not support pollinators showed a higher proportion of synthetic pesticide use within the pesticide-user group.

## Technical Validation

The questionnaire was translated into the target languages by collaborators who were native speakers. To ensure that no differences occurred across the questionnaires other than language, all forms were initially created by the project leader. Editing rights were then granted to collaborators so that they could thoroughly review the wording. Where necessary, they adapted questions by adding country-specific examples to ensure cultural relevance. For clarifications regarding terminology, collaborators consulted with the project coordinator to agree on the most appropriate translation of technical terms. Prior to dissemination, editing rights were restricted to the project leader alone. This procedure was essential to maintain standardisation across all versions, as Google Forms can be modified at any time, potentially resulting in unintended alterations. By exporting each questionnaire from Google Forms according to its language, the surveys were suitable for centralised data management.

All data exported directly from Google Forms were automatically saved into text-based tables (.txt) that preserved both the original submission order and the exact timestamps of responses.

Data quality was assessed through a series of automated and manual validation procedures. For each variable, data type, allowable values, missing values, and consistency across language versions were verified. Categorical variables were checked against the predefined response options, while numerical variables were screened for implausible values and outliers. The merged dataset was then compared with the original exports from Google Forms to confirm that all records and variables had been transferred correctly. Since the termination of the survey, the data were analysed in a variety of ways and used in publications (e.g. Varga-Szilay, *et al*., 2024; Varga-Szilay & Pozsgai, 2022^15,17^ with no obvious data-related weaknesses.

## Usage Notes

Our database provides a unique resource for investigating gardeners’ attitudes, motivations, knowledge, and management practices across nine European countries, where no comparable survey at this scale has previously been conducted. Combined methods of machine learning and network analysis can be applied to explore associations between gardening practices and socio-demographic characteristics (e.g. Varga-Szilay, *et al*., 2024^15^). The data can be reused for cross-country comparisons across the nine participating countries, but it is also possible to extract country-specific subsets, enabling in-depth analyses at the country level as well as pinpointing national characteristics of garden structures, gardening habits or pesticide use. Potential applications also include analyses focusing on pesticide-related gardening practices, which may contribute to a better understanding of usage patterns and support the promotion of alternatives that reduce the reliance on widely available synthetic plant protection products. The dataset also allows researchers or policy makers to identify broader gardeners’ behavioural and attitudinal patterns that may inform future interventions aimed at engaging gardeners and encouraging biodiversity-friendly practices.

Furthermore, population statistics (e.g. Eurostat, 2022, 2024^31,32^) can be integrated with this dataset, enabling normalisation of responses by national or regional population size; for instance, by expressing respondent numbers per 100000 inhabitants in each NUTS-2 region^16^. This compatibility facilitates the inclusion of our dataset both in large-scale comparative analyses and NUTS-level regional studies.

By providing translations of all questions and answer options into the official languages of the nine surveyed CEE countries, in addition to the English version, we ensured that the questionnaire can be readily reused in future studies. This approach facilitates direct replication or partial reuse of the survey in other European regions, including Western and Central-Eastern European regions or the Balkans, in a fully comparable form. Such harmonisation enables cross-country comparisons and supports the development of a unified framework for studying gardening practices and biodiversity-friendly behaviours across Europe.

The dataset does not include precise geographical coordinates of individual gardens and is therefore not suitable for fine-scale spatial analyses. In addition, it does not contain direct measurements of garden biodiversity. Future studies could combine similar questionnaire-based datasets with citizen science initiatives or standardised biodiversity surveys to link gardening practices with observed patterns of biodiversity.

## Data Availability

All data are openly available in the Figshare repository21, under a CC-BY 4.0 Licence. All related computer code is also available on the project’s GitHub repository: https://github.com/zsvargaszilay/Central_Eastern_Europe_Gardening_Dataset.

## Code availability

The computer codes (Python and R) and packages used to clean the dataset and generate the table and the figures in this paper are openly available in the https://github.com/zsvargaszilay/Central_Eastern_Europe_Gardening_Dataset GitHub repository.
